# Movement amplitude on the Functional Re-adaptive Exercise Device: deep spinal muscle activity and movement control

**DOI:** 10.1007/s00421-017-3648-3

**Published:** 2017-05-23

**Authors:** A. Winnard, D. Debuse, M. Wilkinson, L. Samson, T. Weber, Nick Caplan

**Affiliations:** 1 Faculty of Health and Life Sciences, Northumbria University, Northumberland Building, Newcastle upon Tyne, NE1 8ST UK; 2 Space Medicine Office, European Astronaut Centre, European Space Agency, Cologne, Germany

**Keywords:** Motor control, Lumbar multifidus, Transversus abdominis, Posture, Spinal stability

## Abstract

**Purpose:**

Lumbar multifidus (LM) and transversus abdominis (TrA) show altered motor control, and LM is atrophied, in people with low-back pain (LBP). The Functional Re-adaptive Exercise Device (FRED) involves cyclical lower-limb movement against minimal resistance in an upright posture. It has been shown to recruit LM and TrA automatically, and may have potential as an intervention for non-specific LBP. However, no studies have yet investigated the effects of changes in FRED movement amplitude on the activity of these muscles. This study aimed to assess the effects of different FRED movement amplitudes on LM and TrA muscle thickness and movement variability, to inform an evidence-based exercise prescription.

**Methods:**

Lumbar multifidus and TrA thickness of eight healthy male volunteers were examined using ultrasound imaging during FRED exercise, normalised to rest at four different movement amplitudes. Movement variability was also measured. Magnitude-based inferences were used to compare each amplitude.

**Results:**

Exercise at all amplitudes recruited LM and TrA more than rest, with thickness increases of approximately 5 and 1 mm, respectively. Larger amplitudes also caused increased TrA thickness, LM and TrA muscle thickness variability and movement variability. The data suggests that all amplitudes are useful for recruiting LM and TrA.

**Conclusions:**

A progressive training protocol should start in the smallest amplitude, increasing the setting once participants can maintain a consistent movement speed, to continue to challenge the motor control system.

**Electronic supplementary material:**

The online version of this article (doi:10.1007/s00421-017-3648-3) contains supplementary material, which is available to authorized users.

## Introduction

Although somewhat simplified, the maintenance of spinal robustness to ensure static and dynamic stability (Reeves et al. 2007) requires the interaction between two key muscle systems: the short deep muscles that act at a segmental level to modulate spinal stiffness (Hodges 1999, 2004; Hodges et al. 2005b; Hodges and Richardson 1996) and optimal alignment (Claus et al. 2009), and the superficial lumbo-pelvic muscles that generate movement through torque generation, as well as stiffening the spine through co-contraction (Hodges 2004; Hodges et al. 2013b). Panjabi (1992a, b) identified reduced robustness at inter-segmental spinal levels as a key source of low back pain (LBP). Two deep lumbo-pelvic muscles have received particular research attention in this context: lumbar multifidus (LM) and transversus abdominis (TrA). There is evidence that LM provides segmental stiffness (Wilke et al. 1995; Panjabi 1992a), and increases robustness of the spine when stability is challenged (Kiefer et al. 1998), controls the lumbar lordosis (Claus et al. 2009), and makes an important contribution to proprioception (Brumagne et al. 2000). Transversus abdominis contributes to segmental spinal robustness by increasing intra-abdominal pressure (Hodges et al. 2005a). Dysfunction in TrA is associated with dysfunction in LM (Hides et al. 2011), and there is now a substantial body of evidence that links LBP with LM and TrA dysfunction (Hides et al. 2011; Hodges et al. 2006, 2009; Hodges and Moseley 2003; Macdonald et al. 2009; Wallwork et al. 2009).

Importantly, LM function does not return to normal following resolution of LBP (Hides et al. 1996; Macdonald et al. 2009) and this loss of function is considered a likely cause of recurrent episodes of back pain (Hodges et al. 2009; Hodges and Moseley 2003). Lack of cognitive ability to contract the LM and TrA is often considered clinically as a marker of poor motor control used to identify individuals who might benefit from interventions that improve recruitment of these key muscles (Hodges et al. 2013c; Lee 2011; Richardson et al. 2004; Whittaker 2007). However, many healthy people have difficulty cognitively recruiting LM, in particular (Van et al. 2006), which presents a challenge to physiotherapists using interventions that try to activate the muscle. Therefore, an exercise that automatically recruits the LM and TrA in a functional training exercise, might be useful in a LM and TrA rehabilitation context, to facilitate training of the muscles (Debuse et al. 2013; Caplan et al. 2014).

It has previously been argued that interventions for LM and TrA should aim to recruit them in a more tonic than phasic pattern, at low levels of maximal voluntary contraction, to support low-level continuous contractions needed for maintenance of posture (Richardson et al. 2004). Typical exercises that are used early in their rehabilitation involve relatively static tasks, such as the abdominal drawing in manoeuver (Teyhen et al. 2005) and swelling of LM during sitting or lying (Van et al. 2006). However, these tasks often lack functional relevance to more dynamic activities that patients perform in daily life, and the importance of functional therapeutic exercise has been suggested by Hodges and Cholewicki (2007). Any such functional exercise must consider the need to promote tonic, low level activity (Richardson and Jull 1995).

Debuse et al. (2013) investigated a new exercise device, the Functional Re-adaptive Exercise Device (FRED) that aims to recruit LM and TrA muscles. FRED exercise constitutes a combination of weight-bearing, an unstable base of support (at the feet), an upright posture with a relatively robust lumbo-pelvic area, functional lower-limb cyclical movement and real-time visual feedback of performance. This requires the participants’ rearward leg to work eccentrically to control the downward movement of the forward leg, to achieve a smooth, controlled, cyclical motion. The user aims to perform this movement with minimal variability in movement speed. FRED exercise has been shown to promote tonic activity of LM (Caplan et al. 2014; Weber et al. 2017), and to increase lumbo-pelvic robustness when compared to over-ground walking (Gibbon et al. 2013). FRED exercise also promotes similar lumbar lordosis to the ‘short lordosis’ definition from Claus et al. (2009) in people with and without LBP, which would suggest that it is able to recruit LM, even in a clinically relevant population (Winnard et al. 2017) that often has altered activity and size of this muscle (Hides et al. 1994, 2008a; Danneels et al. 2000). As part of ongoing mechanistic studies of the device, the various amplitude settings available during FRED exercise need investigation. This is to inform settings decisions in future studies that will investigate the direct effect of FRED in individuals with impaired motor control and for potential future clinical use of the device.

This study aimed to determine the influence of the amplitude of lower limb motion during FRED exercise on TrA and LM muscle activity and movement variability, to inform the development of an evidence-based FRED exercise prescription.

## Methods

### Participants

Eight-healthy-male participants took part in this study. They had a mean ± SD age, height and mass of 23 ± 6 years, 1.79 ± 0.06 m, and 75.9 ± 7.0 kg, respectively. Exclusion criteria included being aged under 18 or over 55 years, having a history of neuro-musculoskeletal problems or injuries affecting the ability to move (including LBP in the past six months), having heart disease, or having had abdominal or spinal surgery in the last three years. Additionally, participants were required to complete the Physical Activity Readiness and General Practice Physical Activity questionnaires prior to testing. The study was approved by the institutional ethics committee and all participants gave fully informed written consent to take part.

### Protocol

**Fig. 1:**
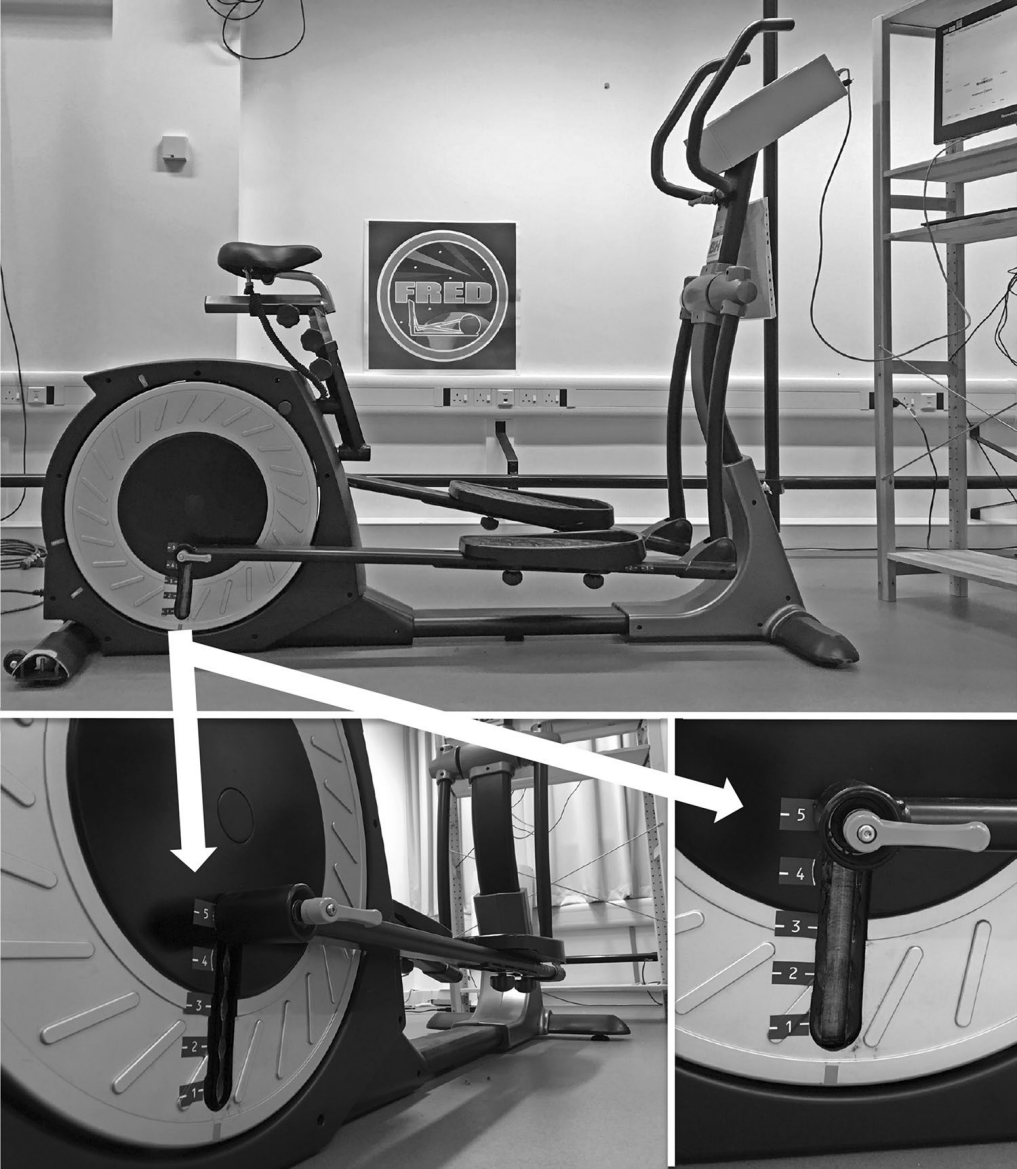
FRED (*top*) and a close up view of the adjustable amplitude crank with the various amplitude settings (*1*–*5*) (*bottom left* and *right*). The crank location is highlighted with a *white arrow*

The FRED has five different amplitude settings, which adjust the distance of the foot plate arm attachment away from the crank axle, with setting one being the largest and five the smallest (Fig. 1). This results in foot plate amplitudes ranging between 0.2 m (setting 5), 0.28 m (setting 4), 0.36 m (setting 3), 0.43 m (setting 2), and 0.5 m (setting 1). Participants exercised, in an upright-standing position, on the FRED at four amplitude settings (settings 2–5), without using the handlebars. Setting one was not used for this study, as pilot investigations found it too difficult for first-time device users to control safely. Participants were given a 5-min familiarisation period exercising on the device in the smallest amplitude setting. Explanation was given of the FRED’s visual feedback in relation to a target frequency and evenness of movement. Participants were allowed an additional 1-min familiarisation period in each amplitude, and the order was randomised for each participant.

The frequency of motion was adjusted for each amplitude to achieve consistent absolute foot speeds that would equate to a constant horizontal speed of 0.42 ms^−1^ (comparable to very slow walking). The target frequency was scaled according to the Strouhal equation (frequency × amplitude/velocity), which has been shown to be within a range of 0.2–0.4 for highly efficient “cruising” locomotion in a large number of animal species (Taylor et al. 2003), and has been proposed to also have relevance to human locomotion (Alexander 2003). By setting the Strouhal number at 0.2 for all amplitudes, it was hypothesised that the calculated target frequency at each amplitude would promote optimal muscular action during FRED exercise.

In addition to the four amplitude settings, LM and TrA thickness at rest (participants lying fully supported on a plinth in a relaxed state) were assessed to normalise absolute LM and TrA thickness data. For LM, this was in prone, with a pillow placed under the abdomen, if needed, to reduce excessive-lumbar lordosis. For TrA, this consisted of crook lying with the knees in 90 degrees of flexion. In all participants, rest was assessed at the start of the testing session, followed by the four randomised-amplitude conditions.

### Data collection

**Fig. 2:**
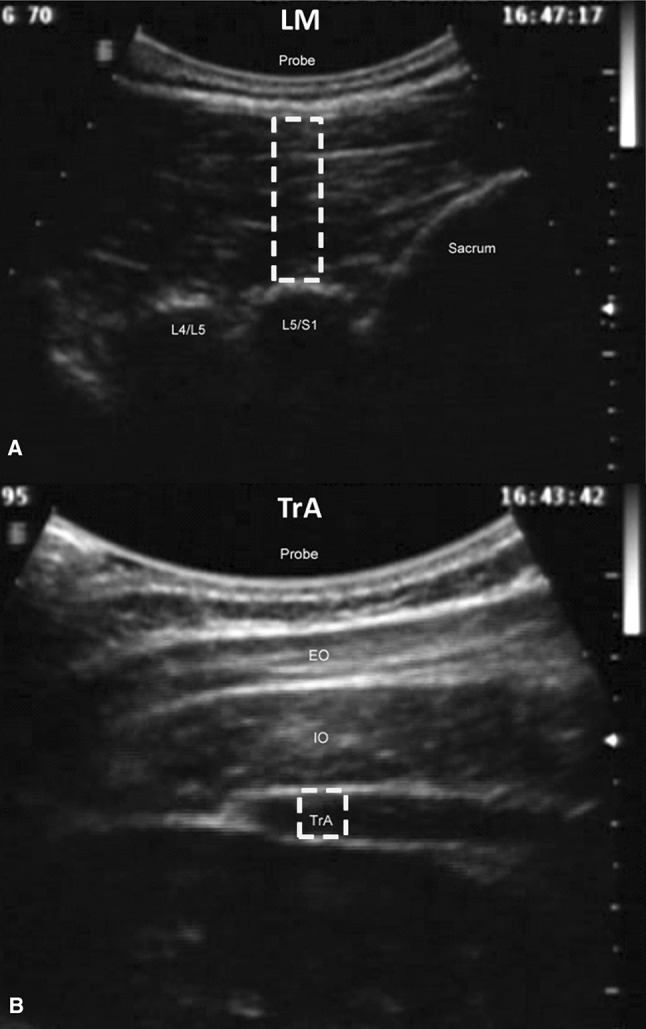
Typical LM (**a**) and TrA (**b**) ultrasound image; the probe, sacrum, L5/S1 and L4/L5 facet joints are labelled. The *white rectangle* shows the location where the area of interest was positioned for automatic edge detection

Muscle activity was assessed by measuring muscle thickness change via ultrasound imaging (USI) in B mode using a digital ultrasound imager with a 2–7 MHz curvilinear transducer (Technos, Esaote, Genoa, Italy). Five seconds of rest and six movement cycle video sequences were recorded continuously in each amplitude setting, for each muscle. Images were adjusted manually to ensure optimum visualisation of the muscles during acquisition. For LM, the transducer was placed longitudinally along the spine with the image midpoint at the L5/S1 facet joint. Thickness was taken as the distance from the echogenic tip of the facet joint to the subcutaneous fascia, based on methods from Kiesel et al. (2007) (Fig. 2a). For TrA, the transducer was placed transversely against the anterolateral abdominal wall, in line with the navel, and the muscle belly was positioned centrally on the image. Thickness was taken as the distance between the upper and lower muscle fascia at a point at least 15 mm lateral to where the muscle tip joined the abdominal aponeurosis, based on methods from Koppenhaver et al. (2009) (Fig. 2b).

**Fig. 3:**
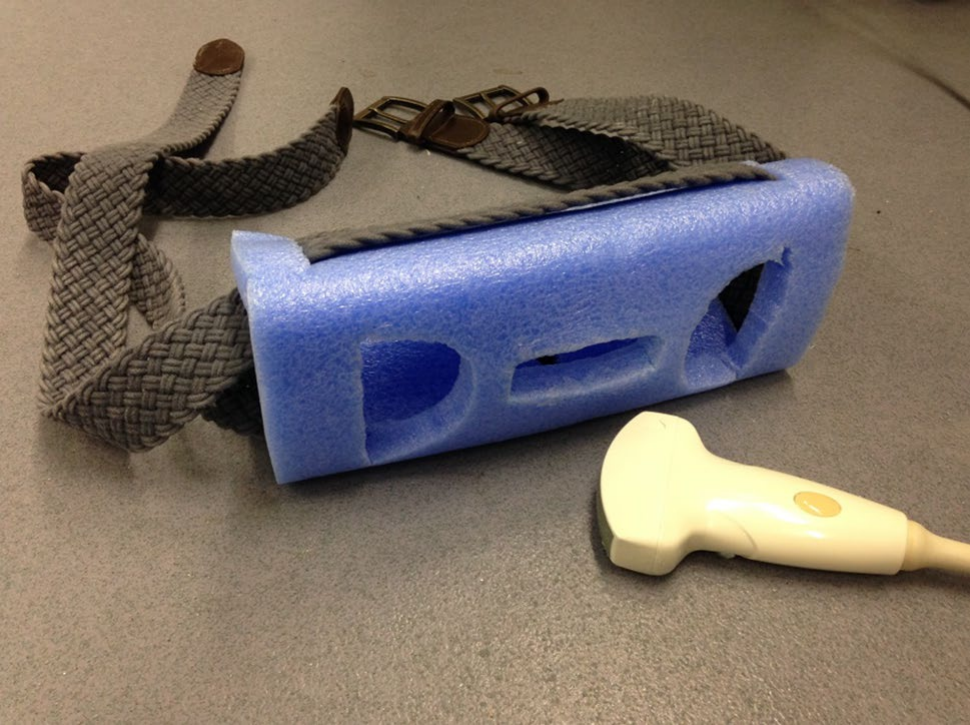
Custom made ultrasound transducer holder and straps

Due to the dynamic nature of the exercise trials, and the need to maintain a constant USI transducer position to obtain video sequences suitable for processing, a bespoke transducer holder (Fig. 3) was used to provide additional support to the researcher who, themselves, held the transducer throughout data collection. The holder consisted of a foam block with a rectangular slot into which the transducer was inserted, which was then secured onto each participant using two adjustable material straps. This allowed the transducer to move with participants’ natural movements. Two holes either side of the rectangular slot allowed additional ultrasound gel to be inserted without needing to remove the holder or transducer.

Periods of ultrasound data were captured at 25 frames per second, at a resolution of 720 × 480 pixels, from a PC connected to the ultrasound imager using a PC-to-TV splitter (SA235, Kworld, CA, USA) and converted from analogue USI output to digital PC input (G5, Terratec, Alsdorf, Germany). The USI video data were recorded in video editing software (MAGIX Video Easy version 3.0.1.5, Terratec, Alsdorf, Germany).

Muscle thickness data were measured using automatic-edge-detection software for USI measurements (Vasculometer 1.2; Bremser et al. 2012). Within the edge-detection software, horizontal and vertical smoothing was set to 3.5 and 10, respectively, near and far wall settings were adjusted to select the facet joint tip and subcutaneous fascia for LM (Fig. 2a), and the near and far fasciae for TrA (Fig. 2b). The smoothing settings refer to a system of reducing noise to create a clear image and with defined structure edges for automatic detection and measurement. The smoothing function overlays adjacent frames and calculates a mean from the overlay to create a single, clearer image. Higher smoothing results in more reliable edge detection, but reduces accuracy due to averaging across more frames. The settings were kept the same throughout all data collection to prevent changes from potentially confounding results. Any sections of video which did not allow adequate visualisation of the muscles were masked and excluded from analysis.

For LM, thickness was measured automatically with the edge-detection software, as the area of interest for analysis was stable throughout the video. In TrA analysis, the lateral movement of the muscle seen during its activation prevented fully automatic analysis as the software was unable to laterally track the area of interest. To overcome this, the area of interest in TrA videos was repositioned manually every five frames to correct for lateral muscle movement and the automatic thickness measurement from Vasculometer manually noted each time. All muscle thickness data were imported into Microsoft Excel 2010 for analysis.

A second individual operated the ultrasound video capture and FRED software. The second individual observed the number of FRED cycles recorded and made a mark on the USI data to show when the start of each cycle occurred.

A key component of FRED exercise is the need to achieve smooth and controlled motion of the lower limbs within each cycle. For this reason, the device was instrumented to record the angular velocity of the crank using a rotary encoder (RP6010, ifm Electronic GmbH, Essen, Germany). Movement variability was quantified as the difference (%) between the instantaneous-angular velocity of movement and the mean-angular velocity over the previous second. This was recorded as a negative change if the live velocity was decreasing and positive if it was increasing. Movement variability data were made absolute for analysis, meaning a high movement variability value indicated uneven movement while a movement variability of zero represented perfectly even movement (i.e. constant angular velocity). It was assumed that a high movement variability result was an indicator of poor motor control, or a more challenging device setting for consideration in a progressive rehabilitation intervention. The movement variability data were recorded at 5 Hz on a second PC, running bespoke FRED software (Mazur Automation, Munchen, Germany). The data were imported into Microsoft Excel 2010 for analysis.

### Data analysis

Magnitude-based inference (MBI) was used to run multiple-pairwise comparisons between the various amplitudes for each variable. These statistics provide the probability (for each comparison) that the true (population) change is positive, negative or trivial with reference to a pre-determined minimal-worthwhile change. This allows an inference on how meaningful any population difference is (Batterham and Hopkins 2006). In the absence of previously reported and validated minimal clinically meaningful change, the standardised mean difference (Cohen’s *d*) was calculated between each comparison by:

$$d = \frac{{{\text{sample mean }}1 - {\text{sample mean }}2}}{{{\text{pooled standard deviation of sample }}1 \, {\text{and }} 2}}.$$

As this study was mechanistic and assessed for an effect between FRED settings, a standardised-mean difference of 0.2 was set as the minimal-worthwhile change on which to base inference as showing at least a small change (Hopkins et al. 2008). In comparisons where variation made small inferences unclear, the worthwhile-change threshold was increased to the lowest level that produced a clear result, of either 0.6 or 1.2 (i.e. moderate or large changes), respectively (Hopkins et al. 2008).

The standardised-mean change, 90% confidence intervals and probabilities (%) that the true values were mechanistically positive, trivial or negative in relation to the minimal-worthwhile change were then reported and defined as <0.5% is “most unlikely”, <5% is “very unlikely”, <25% is “unlikely”, 25–75% is “possibly”, >75% is “likely”, >95% is “very likely”, and >99.5% is “most likely”. Mechanistic inferences are based on threshold chances of 5% for substantial magnitudes (Hopkins et al. 2008). Inferences that were at least ‘likely’ were highlighted in the results.

## Results

### Lumbar multifidus normalised muscle thickness

**Fig. 4:**
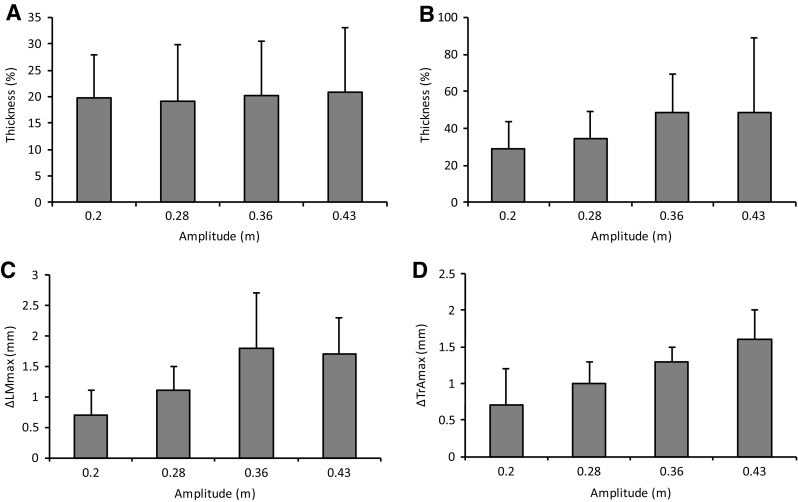
Absolute muscle thickness as a function of amplitude and at rest is shown for LM (**a**) and TrA (**b**), as well as relative change in muscle thickness from rest for LM (**c**) and TrA (**d**)

Figure 4a shows no obvious change in LM thickness between all amplitudes. Magnitude-based inference statistics are reported in Table 1, which show the only *likely* differences between the amplitudes were trivial.

**Table 1:** Difference normalised muscle thickness and muscle thickness variability for LM and TrA, as well as movement variability, between conditions, calculated with threshold for inference of at least 0.2 standardised mean change (unless indicated otherwise)

Movement amplitude	Standardised change in mean	90% Confidence limits		Mechanistic inference
Normalised LM				
0.43–0.36 m	0.1	−0.3	0.4	Very likely trivial^a^
0.43–0.28 m	0.1	−0.1	0.4	Possibly positive
0.43–0.2 m	0.1	−0.3	0.4	Very likely trivial^a^
0.36–0.28 m	0.1	−0.2	0.4	Unlikely positive
0.36–0.2 m	0.0	−0.4	0.5	Very likely trivial^a^
0.28–0.2 m	−0.1	−0.5	0.4	Likely trivial^a^
Normalised TrA				
0.43–0.36 m	0.0	−0.4	0.4	Very likely trivial^a^
0.43–0.28 m	0.3	0.0	0.7	Likely positive
0.43–0.2 m	0.5	0.1	0.9	Likely positive
0.36–0.28 m	0.4	−0.1	0.8	Possibly positive
0.36–0.2 m	0.5	−0.1	1.0	Likely positive
0.28–0.2 m	0.1	−0.2	0.5	Very likely trivial^a^
ΔLM_max_				
0.43–0.36 m	0.4	−0.5	1.3	Possibly positive^a^
0.43–0.28 m	0.4	−0.3	1.0	Possibly positive^a^
0.43–0.2 m	0.4	−0.1	0.8	Likely positive
0.36–0.28 m	−0.1	−0.9	0.7	Very likely trivial^b^
0.36–0.2 m	1.0	0.3	1.7	Very likely positive
0.28–0.2 m	0.8	0.0	1.6	Likely positive
ΔTrA_max_				
0.43–0.36 m	0.5	−0.4	1.3	Possibly positive^a^
0.43–0.28 m	1.3	0.5	2.1	Very likely positive
0.43–0.2 m	1.6	0.9	2.3	Most likely positive
0.36–0.28 m	0.9	0.1	1.7	Likely positive
0.36–0.2 m	1.4	0.6	2.3	Very likely positive
0.28–0.2 m	0.8	0.0	1.6	Likely positive
Movement variability				
0.43–0.36 m	−0.2	−0.7	0.4	Unlikely negative^a^
0.43–0.28 m	1.1	−0.1	2.2	Likely positive
0.43–0.2 m	1.9	0.7	3.1	Very likely positive
0.36–0.28 m	0.9	−0.2	2.0	Likely positive
0.36–0.2 m	1.5	0.2	2.7	Likely positive
0.28–0.2 m	0.7	−0.1	1.5	Likely positive

^a^Inference threshold of 0.6

^b^Inference threshold of 1.2

### Transversus abdominis normalised muscle thickness

Figure 4b shows a trend of increased muscle thickness as the amplitude increased in size. The magnitude-based inference statistics reported in Table 1 shows that increasing the amplitude was *likely* to increase normalised TrA thickness between the two largest and the two smallest amplitudes. However, only a possible trend was observed between the other amplitudes, and became *very likely* trivial between the two largest and two smallest amplitudes, respectively.

### Lumbar multifidus muscle thickness variability

In the smallest amplitude, ΔLM_max_ was 1.1 ± 0.4 mm. It increased to 2.5 ± 2.1 mm in the largest amplitude (Fig. 4c). The magnitude-based inference statistics reported in Table 1 shows high levels of variation across participants resulting in few clear inferences at the 0.2 standardised mean change levels. Larger amplitudes were at least *likely* to result in increased ΔLM_max_ compared to the smallest. However, only a *possible* trend was observed between the other amplitudes, and was trivial between the two smallest amplitudes.

### Transversus abdominis muscle thickness variability

In the smallest amplitude, ΔTrA_max_ was 1.0 ± 0.3 mm. It increased to 1.9 ± 0.6 mm in the largest amplitude condition (Fig. 4d). The magnitude-based inference statistics shown in Table 1 shows that it was at least *likely* that larger amplitudes resulted in increased ΔTrA_max_ except between the two largest amplitudes where the trend was only *possibly* positive.

### Movement variability

**Fig. 5:**
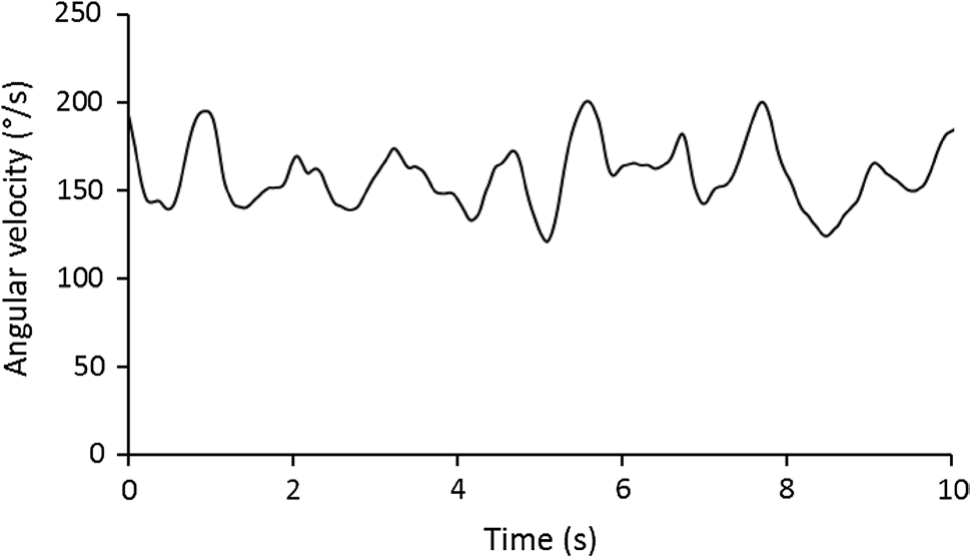
Example angular velocity data shown over a 10 s period for an example participant exercising in the smallest amplitude setting. In this example, the mean angular velocity and rotational frequency were 158.0°/s and 0.44 Hz, respectively

**Fig. 6:**
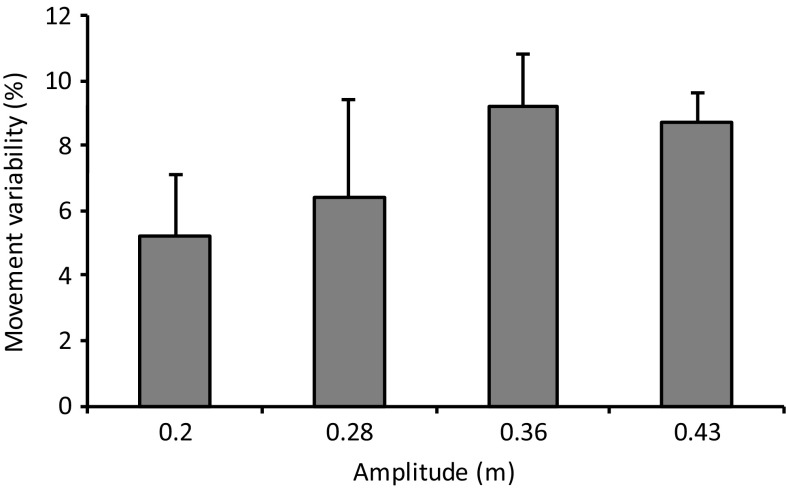
Movement variability is shown as a function of movement amplitude

Figure 5 shows example movement angular velocity data for one participant, from which movement variability was calculated. In the smallest amplitude, movement variability was 5.2 ± 0.9%. It increased to 9.2 ± 3% in the 0.28 m amplitude setting, reducing slightly to 8.7 ± 1.9% in the largest amplitude (Fig. 6). Table 1 shows that it was at least *likely* that larger amplitudes caused increased movement variability. However, the change was *unlikely* negative between the two largest amplitudes.

## Discussion

The main finding of this study was that increasing the movement amplitude while exercising on the FRED resulted in increased variability of lower limb movement. This was linked to increased TrA and LM muscle thickness variability and increased TrA muscle thickness. The data suggest that larger amplitudes increased the challenge placed on the motor control system, making it harder to achieve a controlled-cyclical lower limb movement.

The increased challenge to maintaining controlled-cyclical lower limb movement and greater variability in muscle thickness in larger amplitude settings might be caused by a higher vertical descent through which the forward foot must be controlled by muscle actions of the rearward leg. Increasing the amplitude has the effect of increasing both the distance and the time over which the forward-foot descent occurs. The increased distance is likely to increase the demands on the deep spinal muscles and general motor control of the entire movement.

Traditional LM and TrA training interventions recommend progressive programmes, beginning with isolating muscle recruitment, then recruiting during upright-functional positions while maintaining lumbar lordosis and thoracic kyphosis, before the muscles are trained to work in functional activities, and finally building endurance of the LM and TrA muscles (Hides et al. 2008b; Hodges et al. 2013a, b). As FRED exercise already incorporates an element of functional lower limb movement (Caplan et al. 2014; Gibbon et al. 2013), and is known to promote LM and TrA activity (Debuse et al. 2013), a progressive training protocol using the FRED is likely to begin with the recruitment of muscles, while maintaining lumbar lordosis and thoracic kyphosis, subsequently advancing to training muscle endurance. Recently, Winnard et al. (2017) showed similar spinal kinematics (‘short lordosis’) during FRED exercise between those with and without LBP. Weber et al. (2017) recently found that FRED exercise promotes increased tonic activity of LM and TrA, and reduced activity of the more superficial paraspinal muscles (OI, OE, ES) in asymptomatic participants. Whilst these findings suggest that FRED exercise promotes optimal paraspinal motor control, in line with the specific motor control theory (Hides et al. 2011; Hodges et al. 2006, 2009; Hodges and Moseley 2003; Macdonald et al. 2009; Wallwork et al. 2009), further research is needed to determine how FRED exercise influences paraspinal motor control in people with LBP.

Traditional LM and TrA training progresses the functional movement and endurance stages by reducing base of support, increasing movement size or using physical loading (Hides et al. 2008b; Hodges et al. 2013a, b). As the results of this study show that increasing movement amplitude resulted in increased TrA and LM thickness variability, as well as a reduced ability to maintain even movements, it appears that FRED exercise progression can be based on increasing amplitude size to raise the motor control demand. This progression is also likely to enhance TrA muscle activation. It is, therefore, suggested that users begin in the smallest amplitude setting and increase by one setting when they can maintain a consistent movement speed. Over a period of training, the exercise can be progressed as the user becomes able to control their movement while using a larger amplitude setting that provides an increased motor-control challenge. Further research is needed to evaluate the role of FRED exercise as part of a rehabilitation intervention for LBP as proposed here. Assessing the time taken to reach, and also maintain, good-upright spinal posture with consistent-target-movement variability would also be useful in establishing FRED training protocols.

### Limitations of the study

There was high variability in ΔLM_max_, resulting in larger inference thresholds being used to obtain clear MBI. On closer inspection of the raw data, it was apparent that some participants showed muscle thickness trends much more strongly than others. Further refinement of the USI video data collection for LM may help improve sensitivity and reliability. Movement variability USI data were synchronised manually in this study. Whilst potential errors in doing this are likely to be small due to averaging data over complete movement cycles, future studies should attempt to use improved synchronisation methods. The low sampling rate available from the FRED to record movement variability suggests that the movement variability results should be treated with some caution. Minimal clinically worthwhile changes in relevant outcome measures would also be useful to ascertain and use with MBI if FRED exercise is trialled clinically. In this study, the variability of muscle thickness was determined using ultrasound imaging, as well as the variability of movement. Increases in either of these were assumed to correspond to an increased demand being placed on the motor control system. However, the effects of FRED exercise on motor control were not directly assessed, and this should be considered in future research.

## Conclusion

In conclusion, the FRED recruits TrA and LM more than rest, without the need for voluntary control. Increasing movement amplitude increased the variability of LM and TrA thickness, as well as the variability of lower-limb movement, suggesting an increased challenge to the motor system. Based on this, a training protocol should begin in the smallest amplitude and progress to a larger one once the user demonstrates adequate motor control to exercise with a consistent movement velocity.

## Electronic supplementary material

Below is the link to the electronic supplementary material.

Supplementary material 1 (MOV 31100 kb)

Supplementary material 2 (MOV 25822 kb)

## Abbreviations

FRED: Functional Re-adaptive Exercise Device
LBP: Low back pain
LM: Lumbar multifidus
MBI: Magnitude based inference
TrA: Transversus abdominis
